# The Duffy Antigen Receptor for Chemokines DARC/ACKR1

**DOI:** 10.3389/fimmu.2015.00279

**Published:** 2015-06-05

**Authors:** Richard Horuk

**Affiliations:** ^1^Department of Pharmacology, University of California Davis, Davis, CA, USA

**Keywords:** Duffy antigen, immunology, chemoattractants, antigen receptor, DARC/ACKR1

The discovery that the Duffy antigen was a promiscuous chemokine binding protein was entirely serendipitous and resulted from research begun at Genentech in 1991. The company had a strong interest in chemokines because of their role in disease and this was furthered by the recent cloning of two chemokine receptors CXCR1 and CXCR2. Both were IL-8 (CXCL8) receptors expressed on immune cells, primarily neutrophils, and belonged to the G-protein coupled receptor (GPCR) subfamily, which were highly druggable targets. Numerous reports linked CXCL8 to respiratory diseases like COPD, and Genentech had developed a CXCL8 neutralizing antibody that it thought might be useful therapeutically to treat respiratory diseases like emphysema, bronchitis, and COPD.

Because a number of signal-transmitting polypeptides such as human growth hormone and somatomedin are bound to plasma binding proteins, it was of interest to determine whether blood contained a CXCL8-binding protein. To determine this Walter Darbonne and Caroline Hèbert working in Joffre Bakers lab at Genentech set up a CXCL8 whole blood assay in which increasing amounts of radiolabeled ^125^I-CXCL8 were added to whole blood (1). Interestingly, at low CXCL8 concentrations, the recovery of the chemokine in the plasma was very low but it increased as the CXCL8 concentration was increased. This appeared to be a saturable process and could result from CXCL8 binding to a blood protein. Since erythrocytes are the major cell type in blood, an experiment was set up to examine the effect of adding increasing amounts of radiolabeled ^125^I-CXCL8 to these cells. Using this assay, it was determined that CXCL8 was specifically absorbed by erythrocytes in a saturable manner (1). Furthermore, the erythrocyte-bound CXCL8 was not able to activate the neutrophils by engaging CXCR2 but this process could be reversed by the addition of excess unlabeled CXCL8. The molecule binding the CXCL8 on erythrocytes was a protein because it was sensitive to chymotrypsin treatment; however, trypsin treatment had no effect on binding of CXCL8 (1). Scatchard binding experiments revealed that the binding was saturable and defined by a high affinity receptor, with a binding *K*_D_ of 5 nM and around 2,000 binding sites per red blood cell. Interestingly, it was not always possible to successfully detect CXCL8 binding to blood samples and it appeared that the blood from these CXCL8 non-responders was always from African American donors.

Taking all of this information together, a search of the literature revealed that the CXCL8-binding protein that was discovered at Genentech had molecular properties consistent with those described for a human red blood cell antigen called the Duffy antigen. The Duffy antigen is a human erythrocyte blood group antigen that was shown to be a portal of entry for the malarial parasite *P. vivax* (2). A promoter mutation in the GATA box of the Duffy gene prevents its expression on erythrocytes (3) and most West Africans are resistant to *P. vivax*-*i*nduced malaria because they are homozygous for this mutation (4). To test the hypothesis that the CXCL8-binding protein was the Duffy antigen, we obtained whole blood from Duffy-positive and Duffy-negative donors and showed that there was an absolute correlation of CXCL8 binding to Duffy-positive but not to Duffy-negative blood (5). Further, we demonstrated that the Duffy antigen was a promiscuous chemokine binding protein binding both CXC and CC chemokines (5). Based on these observations, we renamed the Duffy antigen DARC (Duffy antigen receptor for chemokines) (6). Recently, a new nomenclature for atypical non-signaling chemokine receptors such as DARC was adopted and approved and DARC is now known by the acronym ACKR1 (atypical chemokine receptor 1) (7) However, for the purpose of this review, we will stick to the old nomenclature of DARC.

Further work showed that DARC was expressed in other tissues in the body including kidney and brain (8, 9). Around this time, the protein had also just been sequenced (10) and was shown to be a member of the GPCR family. We transfected the newly cloned receptor into K562 cells and were able to recapitulate all of the molecular and functional properties of the protein (11). Furthermore, chemokine binding to DARC blocked both the binding and the infection of human erythrocytes by the malarial parasite *P. vivax* (12).

There is a variety of evidence in support of the idea that DARC on erythrocytes can act as a depot for chemokines reducing their concentration in the circulation (1). In line with this notion, cancer patients undergoing IL-1 immunotherapy were shown to have high erythrocyte CXCL8 binding compared to plasma levels perhaps indicating a potential protective role to prevent chemokine activation of neutrophils and inflammation. In addition, a recent study examining the influence of DARC in kidney transplant rejection in African Americans found that DARC-negative patients had lower allograft survival than DARC-positive patients (13) suggesting to the investigators that perhaps DARC may attenuate the inflammatory effects of chemokines by inactivating them. Further evidence for the protective nature of DARC comes from transfusion experiments in which either DARC wild-type erythrocytes or DARC-negative erythrocytes were transfused into DARC wild-type endotoxemic mice. The mice receiving DARC-negative erythrocytes had increased neutrophil migration into the lungs, increases in inflammatory cytokine concentrations, and increases in lung microvascular permeability compared with mice receiving DARC-wild-type erythrocytes (14). The authors speculated that the pulmonary inflammation that appeared to be induced by a reduction in erythrocyte chemokine scavenging in these experiments could translate to an increase in existing lung inflammation in susceptible Duffy-negative patients.

The idea that DARC can signal in direct response to ligand binding is highly unlikely because although it is a seven-transmembrane-spanning receptor, most members of which are GPCRs, DARC lacks the entire DRYLAIV sequence, a highly conserved determinant of G-protein coupling found in GPCRs at the boundary between the third transmembrane domain and the second intracellular loop. This sequence plays a crucial role in mediating GPCR action and its absence in DARC leads to a failure in its coupling to G-proteins and thus DARC does not mediate a biological signal upon direct chemokine binding.

Of course, there is still a formal possibility that DARC can provide an indirect biological signal in response to chemokine binding. Some support for this idea is provided by the observation that DARC is highly expressed on endothelial cells lining post-capilliary venules (8). The authors speculate that its cellular location could be consistent with a role for DARC in leukocyte trafficking. Arguing against this idea is the fact that individuals lacking expression of DARC on erythrocytes appear to have normal immune function. However, the finding that DARC is expressed on the endothelial cells of both Duffy-negative and -positive individuals (6) suggests that perhaps DARC expression on endothelial cells may be more important for its role in leukocyte trafficking than DARC expression on erythrocytes. The authors speculate that the retention of DARC expression on endothelial cells in Duffy-negative individuals hints at the retention of a possible physiological function in these cells. One feature that characterizes both signaling receptors and chemokine transporters is the internalization of bound ligands. Interestingly, K562 cells transfected with DARC were shown to be able to induce the internalization of radiolabeled chemokines (11). Taken together, these studies suggest that DARC may have a signaling and/or transporter-like role in endothelial cells but that it lacks features associated with direct ligand-activated G-protein receptor signaling.

Evidence suggesting a role for DARC in endothelial cells as a chemokine transporter that can influence leukocyte transmigration comes from studies by Rot and coworkers (15). They have shown that DARC is involved in the transport of chemokines across endothelial cells. This transcytosis of chemokines led to their apical retention but unlike other decoy receptors such as D6 (ACKR2) ligand internalization by DARC did not lead to chemokine degradation. Thus DARC appears to function as an endothelial transporter for chemokine ligands and, leads to chemokine immobilization on apical cell surfaces. It thus appears to play a critical role in leukocyte trafficking. The authors also speculate that DARC on endothelial cells might function as a chemokine rheostat on the blood–tissue interface by supporting the placement and function of suboptimal concentrations of chemokines, but eliminating their excess (16).

The fact that DARC belongs to a family of seven transmembrane domain proteins and that almost 40% of all marketed medicines interact with this class of proteins strongly suggests that DARC is an excellent target for successfully developing therapeutics to treat malaria. A similar approach has already shown benefit in treating AIDS. AIDS is a lethal infectious disease of the immune system caused by the human immunodeficiency virus (HIV), which can be viewed as a paradigm for *P. vivax*-induced-malaria since like its protozoal counterpart, it requires an interaction between an HIV protein, the viral glycoprotein gp120, and either one of the human chemokine receptors CCR5 or CXCR4.

The discovery that chemokine receptors were major coreceptors for HIV entry into the cell prompted a number of pharmaceutical companies to screen for CCR5 inhibitors. The most successful of these is a small molecule inhibitor of the HIV gp120–CCR5 interaction, maraviroc, which is now a registered drug to treat AIDS. An approach to identify small molecule inhibitors of DARC should prove to be of similar benefit in drastically reducing *P. vivax* malaria (Figure 1). Such inhibitors could be a valuable addition to drug combinations that target both *P. falciparum* and *P. vivax* in regions of the world that are endemic for these parasites.

**Figure 1:**
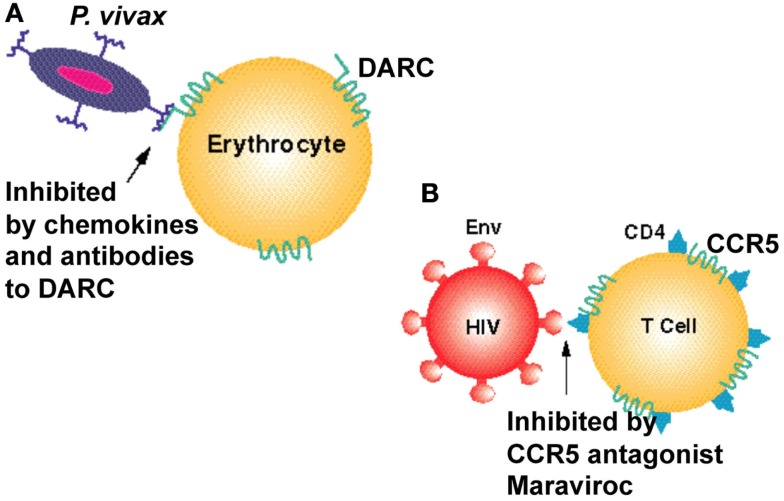
**Seven transmembrane domain receptors are used as vehicles of entry by infectious agents**. **(A)** The human chemokine binding protein DARC is expressed on erythrocytes and is used by *P. vivax* to gain entry to the cell. It can be inhibited by a monoclonal antibody to DARC, Fy6, and by chemokines such as CXCL1 and CXCL8. **(B)** The chemokine receptor CCR5 is used as a co-receptor by the HIV-1 virus to enter human T cells and monocytes. It can be inhibited by chemokines and by the CCR5 inhibitor maraviroc. Cluster of differentiation 4 (CD4) is a glycoprotein expressed on the surface of some immune cells including T lymphocytes. HIV-1 envelope glycoprotein (Env) is responsible for binding to the receptor (CD4) and the chemokine coreceptors (CCR5 or CXCR4) on the host cell, for subsequent fusion of the viral and cellular membranes.

Another interesting parallel between HIV infection and *P. vivax*-induced malaria is that individuals have been identified who are resistant to infection by one or the other of these agents. In the case of HIV, humans with a frameshift mutation in the coding region of the HIV receptor have been identified. This mutation, called delta32, results in the premature truncation of the receptor such that it is no longer expressed. As a consequence, homozygous CCR5 delta32 individuals are resistant to infection by CCR5-tropic HIV strains. These people, who are essentially CCR5 knockouts, do not exhibit any obvious deleterious effects from the lack of this receptor (except for some reports that they are more susceptible to brain infection with West Nile Virus), which may be rationalized by the functional redundancy that seems to be built into the chemokine system where several other chemokine receptors can compensate for a lack of CCR5.

Similarly, individuals in West Africa do not express DARC on their erythrocytes, and they are resistant to *P. vivax*-induced malaria (4). The parallels to the CCR5 delta32 mutation as a protective factor in infection by HIV and the successful development of a CCR5 inhibitor to treat AIDS are striking and we envisage that development of a DARC inhibitor might also work for *P. vivax-i*nduced malaria.

## Conflict of Interest Statement

The author declares that the research was conducted in the absence of any commercial or financial relationships that could be construed as a potential conflict of interest.
